# Long-term outcomes of community-based intensive care treatment following neurological early rehabilitation– results of a multicentric German study

**DOI:** 10.1186/s42466-025-00384-1

**Published:** 2025-05-19

**Authors:** Bernadette Einhäupl, Danae Götze, Stephanie Reichl, Lina Willacker, Romy Pletz, Thomas Kohlmann, Esther Henning, Lena Schmeyers, Andreas Straube, Rebekka Süss, Steffen Fleßa, Simone Schmidt, Jens D. Rollnik, Friedemann Müller, Aukje Bartsch-de Jong, Svenja Blömeke, Jennifer Hartl, Nuria Vallejo, Daniel Liedert, Thomas Olander, Volker Ziegler, Renate Weinhardt, Felix Schlachetzki, Tatjana Groß, Susanne Hirmer, Lea Dillbaner, Lisa Kleinlein, Thomas Platz, Andreas Bender

**Affiliations:** 1 Department of Neurology, LMU University Hospital, LMU Munich, Munich, Germany; 2 Neurorehabilitation Research Group, Faculty of Medicine, University of Greifswald, Greifswald, Germany; 3 Institute for Community Medicine, Department Methods of Community Medicine, University Medicine Greifswald, Greifswald, Germany; 4 Faculty of Law and Economics, Chair of General Business Administration and Health Care Management, University Greifswald, Greifswald, Germany; 5 Institute for Neurorehabilitation Research (InFo), BDH-Clinic Hessisch Oldendorf, Affiliated Institute of Hannover Medical School, Hessisch Oldendorf, Germany; 6 Schön Clinic Bad Aibling-Harthausen, Bad Aibling, Germany; 7 Therapiezentrum Burgau, Hospital for Neurological Rehabilitation, Burgau, Germany; 8 Hospital for Neurological Rehabilitation, Rhön-Clinic, Bad Neustadt a. d. Saale, Germany; 9 Department of Neurology, Centre of Vascular Neurology and Intensive Care, medbo Bezirksklinikum Regensburg, University of Regensburg, Regensburg, Germany; 10 Statistical Consulting StaBLab, Department of Statistics, LMU Munich, Munich, Germany; 11 BDH-Klinik Greifswald, Institute for Neurorehabilitation and Evidence‑Based Practice, University of Greifswald, Greifswald, Germany; 12 Department of Neurorehabilitation, Medical Faculty, University of Augsburg, Augsburg, Germany

**Keywords:** Rehabilitation, Weaning, Survival rate, Tracheal cannula, Mechanical ventilation, Stroke

## Abstract

**Background:**

Weaning from mechanical ventilation (MV) and tracheal cannula (TC) during neurological early rehabilitation (NER) is mostly successful. However, some patients leave NER with TC/MV, requiring home-based specialized intensive care nursing (HSICN). Data on medical and demographic characteristics and long-term outcomes of these patients are limited.

**Methods:**

A multicentric retrospective observational study across five German NER hospitals collected data from neurological patients with TC/MV at discharge. The study aimed to assess patients’ health status at NER discharge, and to identify predictors of post-discharge survival. Survival rates were analyzed using Kaplan-Meier estimates; further predictors of survival post-discharge were analyzed using Cox regression.

**Results:**

Among 312 patients, the one-year survival rate was 61.9%, decreasing to 38.1% after approximately 4 years. Older age, higher overall morbidity and discharge with MV were associated with an increased likelihood of death, while a longer stay in NER correlated with survival.

**Conclusions:**

Patients requiring HSICN after discharge from NER have a high mortality rate. Identifying survival predictors may help to identify patients at risk, and thus could be integrated into the decision-making process for NER discharge. The high mortality post-discharge warrants an evaluation of the current post-hospital care model. Optimizing therapeutic care in the HSICN setting may have the potential to reduce mortality and neuro-disability, and enhance the quality of life in these neurologically severely affected patients.

**Trial registration:**

The trial *OptiNIV - Retrospective study of post-hospital intensive care in neurological patients* has been retrospectively registered in the German Clinical Trials Register (DRKS) since 28.10.2022 with the ID DRKS00030580.

**Supplementary Information:**

The online version contains supplementary material available at 10.1186/s42466-025-00384-1.

## Background

Common neurological and neurosurgical diseases, such as stroke, traumatic brain injury (TBI), or hypoxic-ischemic brain injury (HIE), may be accompanied by disorders of consciousness (DoC), respiratory insufficiency, and dysphagia with risk of aspiration pneumonia. Frequently, these patients are in need of tracheostomy with a tracheal cannula (TC) to protect the airway and, if necessary, mechanical ventilation (MV) [1]. The majority of patients can be decannulated while on the intensive care unit (ICU), but about 40% are transferred to early neurorehabilitation (NER) treatment with continued need for TC and about 25% with need for MV [2]. In Germany, NER is a specialized part of the acute hospital care setting, where rehabilitative therapies are increasingly administered with at least 300 min of therapy per day as individual combinations of physiotherapy, occupational therapy, speech and language therapy, music therapy, physical therapy, respiratory therapy, activating nursing therapy and neuropsychological therapy. At the same time intensive care treatment options are still available on-site, as needed. There are well over 1,000 beds for MV in an NER setting in Germany, with specialization for weaning from MV and TC [3]. Inpatient NER treatment takes an average of 56 ± 51 days [2]. Weaning from MV and from TC represent two major goals during NER. In Germany, approximately 20% of patients are discharged from NER to the community setting with continued need for TC and 6% with continued need for MV [2]. The necessary care is provided in the form of home-based specialized intensive care nursing (HSICN), located in shared apartments, specialized nursing homes, or a private setting [4]. It is delivered by registered nursing staff and compensated by German health insurances. The number of patients discharged with TC and/or MV to HSICN increased from 16,000 in 2016 [5] to about 20,000 in 2019 [5, 6]. Reasons for increased need for HSICN are multifactorial and include decreasing length of stay in hospitals (including NER), increasing age of patients, and high burden of morbidity [2, 5]. HSICN poses an enormous financial burden for the health care system with costs estimated at approximately four billion € per year [6].

Neurological HSICN patients may have a long-term potential for partial recovery of neurological functioning and participation, including delayed successful weaning from TC and/or MV, provided that there is continuing rehabilitation [7]. It is a highly vulnerable population at high risk of dying and suffering complications. Previous studies showed that one year after discharge to the HSICN setting, only 25% of patients with MV and 50% of patients with TC survived, compared to 85% of patients discharged from NER without TC and/or MV [2, 8]. The presence of a TC also leads to a significantly poorer survival rate in the long term (5 years after discharge) [9]. A meta-analysis of the one-year survival rate after long-term MV across international studies showed a mortality rate of 62% in high-quality studies [10].

Thus, it is crucial to establish clinical pathways and strategies for continued neurological rehabilitation focusing on weaning from TC and/or MV in the HSICN setting in order to improve quality of life (QoL) and participation [4]. To this end, the objectives of this retrospective study were to obtain a detailed overview about the characteristics, health status, and care situation of patients with TC and/or MV in HSICN following an inpatient stay in NER and to examine long-term treatment outcomes as well as survival rates and predictors of survival of this patient group in the existing HSICN standard-of-care.

## Methods

### Study design

This multicentric retrospective observational study collected data on the health status of neurological patients at two points in time: at the end of inpatient NER at discharge to HSICN (retrospective design) as well as a follow-up in the form of a Residents’ Registration Office query.

Patients were identified and enrolled in five large German NER hospitals (Schoen Clinic Bad Aibling Harthausen; Therapiezentrum Burgau; Department of Neurology of the University of Regensburg at medbo district hospital; Department of NER/Intensive Care, Rhön-Klinikum, Campus Bad Neustadt; BDH-Klinik Hessisch Oldendorf). This study was approved by the Institutional Review Board of the Medical Faculty of the University of Munich (no. 21-1091). The study was registered with the German Clinical Trials Register (DRKS) on October 28, 2022 (Study ID DRKS00030580).

### Participants

All medical charts of consecutive patients discharged to the HSICN with a TC/MV between 01.01.2019 and 30.06.2021 were screened for study eligibility, according to the following criteria. Inclusion criteria were [1] age ≥ 18 years; [2] previous inpatient NER treatment in one of the partner clinics with a neurological rehabilitation diagnosis; [3] presence of MV and/or TC at discharge from NER; [4] for follow-up interviews: informed consent for study participation. Exclusion criteria were [1] palliative care/life expectancy < 12 months at discharge from NER; [2] weaning/decannulation medically excluded (due to neuromuscular or tumor diseases causing respiratory failure or obstructed nasal/oral breathing); [3] preexisting HSICN prior to inpatient NER stay in one of the recruiting hospitals; [4] progressive neuromuscular disease.

### Data collection

Patient data were collected at two points in time. The date of the patient’s discharge from NER marked the beginning of the observation period (based on the patient’s medical record; retrospective analysis). For all patients, the date of the Residents’ Registration Office query (26.10.2022) served as the end of the observation period. Endpoints were collected fully anonymized based on the medical records of the discharging NER facility, and status of survival was collected by the electronic Residents’ Registration Office query. Follow-up data were available for approximately 10% of the cohort (see Supplementary Table 1).

### Outcome measures

The following endpoints were obtained from the patient’s medical record: personal data and medical history including duration of diseases (onset of disease until discharge from NER), ventilation, tracheostomy care, duration of NER stay and status and modalities of TC and/or MV. Regarding potential decannulation, five possible reasons for failing decannulation during NER were asked for (invasive ventilation, dysphagia, subglottic or tracheal secretion management, tracheomalacia). The main diagnoses that led to admission to the NER unit were divided into five groups (stroke, HIE, TBI, critical-illness-polyneuropathy/-myopathy (CIP/CIM), other neurological disorders) and documented accordingly. Other neurological disorders included, for example, encephalitis, status epilepticus, polyneuritis, other polyneuropathies, myotonic syndrome, myasthenia gravis, polymyositis, except progressive neuromuscular disease (exclusion criteria). Additional diagnoses relevant to care were collected and were examined for the prevalence of the following significant comorbidities: pulmonary disease (including pneumonia), obesity, epilepsy, disorder of consciousness (DoC), malignant tumor, chronic kidney disease, and type 2 diabetes. The term DoC covers patients in various states of consciousness: coma, vegetative state/unresponsive wakefulness syndrome (UWS) and minimal conscious state [11]. In addition, the extent of care access, consisting of a checklist, neurological status (Glasgow Outcome Scale Extended (GOS-E)) and activities of daily living (Barthel Index, (BI)), was recorded for all patients. The GOS-E is an ordinal scale for assessing neurological status and consists of up to four questions in eight categories. These are used to classify the patient on an eight-point scale from 1 = dead to 8 = no restrictions [12–14]. The BI is an instrument to assess activities of daily living (ADL), ten different areas of activities are addressed with scores of 0, 5, 10 or 15 points. The maximum achievable score is 100 points, and means that a patient is able to eat, move around and perform personal hygiene independently and that all the activities listed can be carried out independently [15].

### Statistical analysis

The distribution of endpoints was analyzed descriptively to characterize the study population at the time of discharge from NER. Demographic and clinical characteristics are presented as frequency and percentage for dichotomous variables and mean ± standard deviation (SD) for quantitative variables. Results from ordinal scaled data are expressed as median and interquartile range (IQR). If main variables were not normally distributed, non-parametric statistical tests were used: comparisons between groups were performed with the Chi-square test for dichotomous variables and the Mann-Whitney-U-test for quantitative variables within two groups. Survival rates were estimated using the Kaplan-Meier method and are shown for 6 months, 1 year, 2 years and 3 years after discharge from NER. Cox regression following the stepwise forward method was performed to identify variables correlating with time to death. The independent variables were selected based on group comparison between survivors and non-survivors if there was a significant difference one year after discharge. Independent variables were further analyzed for multicollinearity before being included in the regression and were excluded when the correlation coefficient was > 0.8. Risk was quantified as hazard ratios along with a 95% confidence interval (CI). A hazard ratio < 1 indicates a reduced likelihood of death, while hazard ratios > 1 reflect an increased likelihood of death [16, 17]. Proportional hazard assumption was tested graphically for possible predictors by plotting Schoenfeld residuals versus survival time, which should show as a random dispersion around zero, if the assumption of proportional hazards holds [18]. Statistical significance was set at *p* <.05. Statistical analyses were performed using IBM SPSS Statistics software (version 29). The study was conducted in accordance with the STROBE guideline for cohort studies; the STROBE checklist can be found in the Supplemental Material (see Supplementary Checklist 1). The datasets analyzed during the current study are not publicly available for reasons of data protection law but are available from the corresponding author on reasonable request.

## Results

### Characteristics of the patient sample

In total, data from 312 patients could be collected (67.6% male, 32.4% female; Table 1). One year after discharge from NER 61.9% (*n* = 193) of the patients were alive, and 38.1% (*n* = 119) patients had died. At this point, the mean survival time among the non-survivors was about 4 months and 15 days (137 ± 112, range 1-165 days). The mean observation period for the entire study sample was about 2.6 years (952 ± 267 days, range 475–1387 days) and did not differ between survivors (963 ± 274) and non-survivors (933 ± 255, *U* = 10736.5, *p* =.334).

**Table 1:** Patient characteristics: baseline data one year after discharge for total study sample and the two subgroups of living and deceased patients

	Total	Survivors	Non-Survivors	*P*-value
N (%)	312 (100)	193 (61.9)	119 (38.1)	
Age, years, mean (SD)	65.5 (12.9)	62.8 (13.7)	70.1 (9.4)	< 0.001*
Male, N (%)**	211 (67.6)	125 (64.8)	86 (72.3)	0.174
Primary diagnosis, N (%)**				
Stroke	140 (44.9)	86 (44.6)	54 (45.4)	0.907
Hypoxic-ischemic brain injury (HIE)	53 (17.0)	37 (19.2)	16 (13.4)	0.216
Traumatic brain injury	45 (14.4)	30 (15.5)	15 (12.6)	0.511
Critical illness polyneuropathy/myopathy	34 (10.9)	19 (9.8)	15 (12.6)	0.459
Other neurological disorders	40 (12.8)	21 (10.9)	19 (16.0)	0.223
Number of significant comorbidities, mean (SD)	1.03 (0.85)	0.87 (0.74)	1.16 (0.92)	< 0.001*
Significant comorbidities, N (%)**				
Pulmonary disease (incl. pneumonia)	120 (38.5)	58 (30.1)	62 (52.1)	< 0.001*
Obesity	20 (6.4)	14 (7.3)	6 (5.0)	0.486
Epilepsy	69 (22.1)	40 (20.7)	29 (24.4)	0.484
DoC	33 (10.6)	27 (14.0)	6 (5.0)	0.013*
Malignant tumor	24 (7.7)	10 (5.2)	14 (11.8)	0.048*
Chronic kidney disease	23 (7.4)	7 (3.6)	16 (13.4)	0.003*
Type 2 diabetes	32 (10.3)	14 (7.3)	18 (15.1)	0.034*
Duration of disease, days, mean (SD)	158 (78)	165 (82)	149 (70)	0.044*
Duration of ventilation, days, mean (SD)	55 (53)	50 (53)	64 (52)	< 0.001*
Time since tracheostomy, days, mean (SD)	132 (62)	137 (68)	124 (52)	0.08
Length of stay in NER, days, mean (SD)	121 (59)	128 (66)	111 (45)	0.01*
Discharge with TC only, N (%)**	280 (89.7)	183 (94.8)	97 (81.5)	< 0.001*
Discharge with TC and MV, N (%)**	32 (10.3)	10 (5.2)	22 (18.5)	< 0.001*
GOS-E, median (IQR)	3 (1)	3 (1)	3 (1)	0.474
- Upper severe disability, Score 4, N (%)**	3 (1)	3 (1.5)	0 (0)	
- Lower severe disability, Score 3, N (%)**	224 (71.8)	136 (70.5)	88 (73.9)	
- UWS, Score 2, N (%)**	85 (27.7)	54 (27.9)	31 (26.1)	
BI, median (IQR)	15 (0)	15 (0)	15 (0)	0.293

* Significant difference between alive and deceased patients

** The percentage given for survivors and non-survivors per variable refers to the total number of survivors or non*-survivors at one year after discharge*

Deficient/insufficient tracheal secretion management (92.6%) and the presence of dysphagia with an existing risk of aspiration (90.4%) were the most frequent reasons why decannulation was not successful during NER. More than half of the patients (63.1%) could not be decannulated due to problems with secretion management. Need for invasive ventilation (10.3%) and the presence of tracheomalacia (6.7%) were less frequent reasons for decannulation not taking place.

### Survival rates after discharge from NER

Survival rates after discharge decreased from 61.9% after 1 year to 41.6% after 3 years (Table 2). By the end of the observation period (day of the query at the Residents’ Registration Office) after approximately 4 years (1385 days), the Kaplan-Meier estimate for cumulative proportion of survival was 38.1%.

**Table 2:** Summary of Kaplan-Meier estimate for the total study sample

Time (years)	Cumulative Proportion Surviving at the Time (%)	Cumulative Number of Deaths (*N*)
0.5	75.3	77
1	61.9	119
2	52.8	145
3	41.6	167
End of Observation	38.1	171

**Fig. 1:**
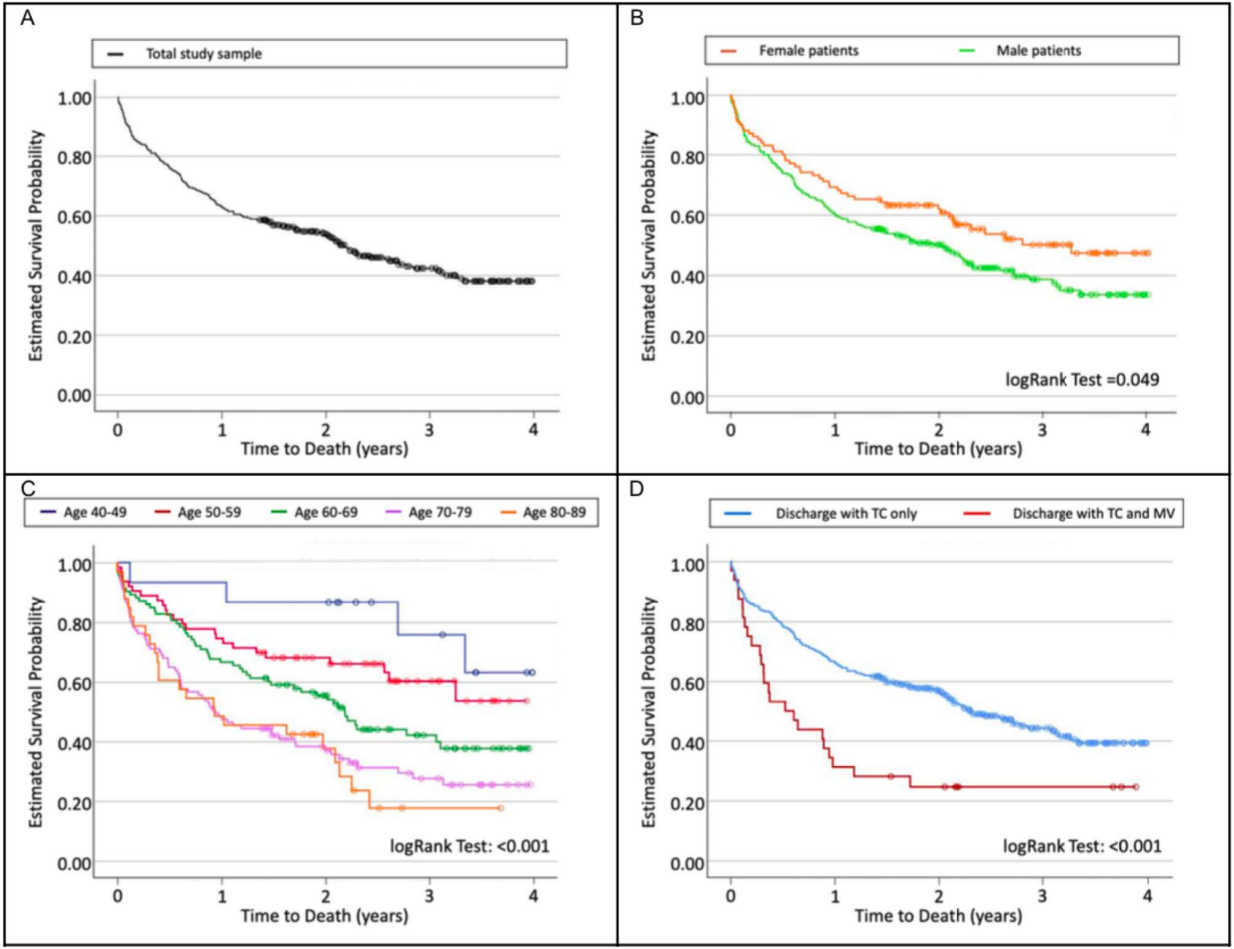
Kaplan-Meier estimates of time to death by (**A**) Total study sample; (**B**) Gender; (**C**) Age groups and (**D**) Discharge with TC only or TC and MV

Survival curves over all patients as well as according to different variables are shown in Fig. 1. Male gender, older age, and need for MV were all associated with increased likelihood of death.

Further, time to death was explored using a Cox proportional hazard model. The analysis revealed that age, number of significant comorbidities, discharge with MV, duration of NER and additional diagnosis of DoC significantly affected time to death (Table 3). Increases in age, number of significant comorbidities, and discharge with MV were associated with a higher likelihood of death. In contrast, increasing length of stay in NER and the presence of DoC as a critical additional diagnosis were associated with a higher likelihood of survival. An analysis of patients with DoC (10.6%) showed that patients with DoC were significantly younger (59.6 ± 15.6) than patients without DoC (66.3 ± 12.2; *U* = 3363, *p* =.011).

**Table 3:** Cox proportional hazard model for significant variables on the risk of death

Variable	Hazard Ratio	*P*-value	95%-CI
Discharge with MV	1.602	0.037	[1.030; 2.492]
Number of significant comorbidities	1.499	< 0.001	[1.252; 1.794]
Age	1.044	< 0.001	[1.028; 1.059]
Duration of NER	0.996	0.022	[0.993; 0.999]
DoC*	0.250	< 0.001	[0.117; 0.537]

*Reference category was set as ´no´ for Discharge with MV and DoC

## Discussion

Our study population has characteristics comparable to previously reported data in terms of age and main diagnoses [2, 9, 19]. Among the study sample, 10.4% required invasive out-of-hospital MV upon discharge from NER, which seems to be in line with current results from a centre for weaning from ventilation [9]. Yet, the data reported here deliberately reflect a negative selection of patients discharged from NER owing to their continued need for TC and/or MV. This is reflected by a high percentage of severe outcomes with more than 70% being completely or almost completely dependent on care (GOS-E score 3), and more than a quarter in a UWS (GOSE-E score 2). According to a Germany-wide, multi-centre study from 2006, about 44.7% of NER patients were discharged with a GOS-E score of 2 or 3 [20].

The high burden of morbidity of neurological patients in HSICN is reflected by the fact that more than one third (38.1%) of all patients had died by one year after NER discharge. Additionally, the mean survival time for these patients was less than 5 months. The Kaplan-Meier estimates for survival rates clearly indicate a higher proportional mortality rate within the first year after discharge with declining dynamics over the years to follow.

As previously reported, neurological patients who are discharged from the NER without TC/MV had a higher survival rate of 85% one year after discharge [2] compared to patients from our study (61.9%). However, the one-year and three-year survival rates (41.6%) are comparable with the ones of patients discharged from weaning centres after prolonged weaning with predominantly internal comorbidities (66.5% and 47.3%) [9]. A group of patients who survived severe infections to septic shock had a better outcome in showing a five-year survival rate of 56.1% [21]. Noteworthy, compared to the situation 10 years ago, the 1-year survival rates reported here have increased (TC: from 50 to 65%; TC + MV: from 25 to 31%) [2], while some progress in healthcare might have occurred in the past decade.

The most important factors for survival after discharge from the NER were determined using Cox regression. They reveal that older age, multi-morbidity and discharge with MV were associated with an increased likelihood of death. The results on advanced age, comorbidities (such as lung disease, chronic kidney disease, type 2 diabetes), and the duration of MV are consistent with previous research. These factors are associated with a higher mortality rate in specialized weaning centers [9, 22]. A longer duration of NER and DoC as an additional diagnosis were associated with a lower probability of death during the reporting period. DoC patients were significantly younger than patients without DoC. An extended model including age, DoC and their interaction term (age*DoC) showed that increasing age was significantly associated with a higher probability of death in the group without DoC. Yet, in the group with DoC, age had little impact on survival probability. The presence of DoC was significantly positively associated with survival in patients of average age (Hazard Ratio = 0.282), but the interaction term showed that this protective effect diminished with increasing age (Hazard Ratio = 0.952). Hence, the model revealed that the statistical effect of DoC on survival depended on age, being more pronounced in younger patients, while age became the dominant factor in older patients (see Supplementary Table 2). In younger DoC patients, the positive association with survival may hypothetically stem from reduced therapeutic interventions post-discharge (e.g., longer deflated TC cuff use, oral nutrition, speaking valves) that would have required active cooperation while at the same time being associated with increased health risks such as pneumonia. The assumption of their more frequent application in non-DoC patients might be considered as potential explanation. However, due to limited HSICN data and absence of related studies, this explanation remains hypothetical.

With outpatient care for critically ill patients, the HSICN offers the opportunity to discharge patients early from NER and thus treat more patients as inpatients. However, a longer NER duration is associated with a higher probability of survival in the HSICN. This underlines the relevance of the NER and its importance for the participation of critically ill neurological patients after discharge. It should be considered that a longer NER could be particularly useful for those patients who exhibit high morbidity and mortality, i.e., older patients with several critical co-morbid diagnoses and a prolonged need for MV.

Key elements of inpatient NER include goal-oriented, interdisciplinary, and frequently adapted therapy carried out by a multi-professional team [23–25]. These elements crucially influence the long-term outcome in NER. Yet, in the community-setting, those key elements are hardly ever achievable. Optimization of the existing HSICN standards, particularly with regard to weaning and decannulation, might therefore be a promising strategy to improve survival rates and neurological outcomes in TC/MV patients. Specific interventions aimed at increasing weaning rates in the community setting should be tested to increase QoL, decrease dependence in the ADL, and potentially reduce the enormous health care costs associated with HSICN. To this end, a multicentric prospective study (OptiNIV; www.optiniv.de) was recently initiated, in which HSICN patients will be visited by specialized expert teams for weaning and decannulation in order to improve weaning rates [4].

The limitations of this study are associated with its retrospective and observational nature. Data of interest (i.e., medical history and status at discharge from NER) had to be obtained from medical records retrospectively, which might affect data quality. However, most of the extracted data was standard routine data from the healthcare sector, which supports its validity. With regard to attrition bias, a high percentage of coverage of data (medical history and status at discharge from NER) for eligible subjects was made possible by integrating anonymous data from deceased persons (when death was ascertained by public registry entries). Conducted in German NER centers, the study’s applicability may be limited in settings with different discharge practices. In Germany, patients who require TC/MV after ICU can be transferred to specialized weaning units within neurorehabilitation ICUs. If decannulation/weaning fails during NER, further care can be provided in HSICN. Despite its limited generalizability, the study offers a detailed overview of medical characteristics, survival rates, and predictors in this model of care.

## Conclusion

It is important for NER inpatient treatment teams to be aware of the high morbidity and mortality of neurological HSICN patients. Hence, particular attention should be paid to potential at-risk groups, such as older, multi-morbid, ventilated patients, when making discharge decisions. In the light of the observed extraordinarily high mortality rate, the presence of one or more of these risk factors might call for a delay of NER discharge and warrants rigorous preparation and quality assurance of the HCSIN teams.

## Electronic supplementary material

Below is the link to the electronic supplementary material.

Supplementary Material 1

## Data Availability

Study data can be analysed and used by the research groups and for purposes agreed on by participants in writing. Any request regarding the dataset supporting the conclusions of this article should be proposed to the authors.

## Abbreviations

ADL: Activities of daily living
BI: Barthel Index
CI: Confidence interval
CIP/CIM: Critical-illness-polyneuropathy/-myopathy
DoC: Disorders of consciousness
GOS-E: Glasgow outcome scale– extended
HIE: Hypoxic-ischemic brain injury
HSICN: Home-based specialized intensive care nursing
ICU: Intensive care unit
IQR: Interquartile range
MV: Mechanical ventilation
NER: Neurological early rehabilitation
QoL: Quality of life
SD: Standard deviation
TBI: Traumatic brain injury
TC: Tracheal cannula
UWS: Unresponsive wakefulness syndrome
