# Supplementary material for: Long-term outcomes of community-based intensive care treatment following neurological early rehabilitation– results of a multicentric German study
Source: Neurol Res Pract. 2025 May 19;7(1):35. doi: 10.1186/s42466-025-00384-1 (PMC12093594; doi:10.1186/s42466-025-00384-1)
Supplement: Supplementary file 1 — Supplementary Material 1 [file 42466_2025_384_MOESM1_ESM.docx]

Supplementary Checklist 1, Additional File 1

STROBE Statement—Checklist of items that should be included in reports of ***cohort studies***

|  | Item No | Recommendation |
| --- | --- | --- |
| **Title and abstract** | 1 | (*a*) Indicate the study’s design with a commonly used term in the title or the abstract DONE (in abstract) |
|  |  | (*b*) Provide in the abstract an informative and balanced summary of what was done and what was found  INCLUDED (in abstract) PAGE 2 |
| Introduction | | |
| Background/rationale | 2 | Explain the scientific background and rationale for the investigation being reported PAGE 4 LINES 90-98 |
| Objectives | 3 | State specific objectives, including any prespecified hypotheses PAGE 4 LINES 99-105 |
| Methods | | |
| Study design | 4 | Present key elements of study design early in the paper PAGE 5 LINES 108-118 |
| Setting | 5 | Describe the setting, locations, and relevant dates, including periods of recruitment, exposure, follow-up, and data collection PAGE 5 LINES 108-111 (for setting) and PAGE 5 LINES 112 -115 (for location) and PAGE 6 LINES 131-136 (for relevant dates) |
| Participants | 6 | (*a*) Give the eligibility criteria, and the sources and methods of selection of participants. Describe methods of follow-up PAGE 5 LINES 121-128 |
|  |  | (*b*) For matched studies, give matching criteria and number of exposed and unexposed N/A |
| Variables | 7 | Clearly define all outcomes, exposures, predictors, potential confounders, and effect modifiers. Give diagnostic criteria, if applicable PAGE 6 LINES 139-162 |
| Data sources/ measurement | 8* | For each variable of interest, give sources of data and details of methods of assessment (measurement). Describe comparability of assessment methods if there is more than one group PAGE 6 LINES 139-162 |
| Bias | 9 | Describe any efforts to address potential sources of bias N/A |
| Study size | 10 | Explain how the study size was arrived at N/A, MAXIMAL RECRUITMENT WITHIN STUDY PERIOD |
| Quantitative variables | 11 | Explain how quantitative variables were handled in the analyses. If applicable, describe which groupings were chosen and why PAGE 7 LINES 165- 172 |
| Statistical methods | 12 | (*a*) Describe all statistical methods, including those used to control for confounding PAGE 7 LINES 169-184 |
|  |  | (*b*) Describe any methods used to examine subgroups and interactions PAGE 7 LINES 175-176 |
|  |  | (*c*) Explain how missing data were addressed N/A |
|  |  | (*d*) If applicable, explain how loss to follow-up was addressed N/A |
|  |  | (*e*) Describe any sensitivity analyses N/A |
| Results | | |
| Participants | 13* | (a) Report numbers of individuals at each stage of study—eg numbers potentially eligible, examined for eligibility, confirmed eligible, included in the study, completing follow-up, and analysed PAGE 8 LINE 192 |
|  |  | (b) Give reasons for non-participation at each stage N/A (retrospective design) |
|  |  | (c) Consider use of a flow diagram INSUFFICIENT INFORMATION |
| Descriptive data | 14* | (a) Give characteristics of study participants (eg demographic, clinical, social) and information on exposures and potential confounders PAGE 8 LINES 192-197 and TABLE 1 |
|  |  | (b) Indicate number of participants with missing data for each variable of interest N/A |
|  |  | (c) Summarise follow-up time (eg, average and total amount) PAGE 8 LINES 195-197 |
| Outcome data | 15* | Report numbers of outcome events or summary measures over time PAGE 10 LINES 213-216 and PAGE 12 LINES 226-233 and FIGURE 1 and TABLE 2 and 3 |
| Main results | 16 | (*a*) Give unadjusted estimates and, if applicable, confounder-adjusted estimates and their precision (eg, 95% confidence interval). Make clear which confounders were adjusted for and why they were included TABLE 1-3 and FIGURE 1 |
|  |  | (*b*) Report category boundaries when continuous variables were categorized N/A |
|  |  | (*c*) If relevant, consider translating estimates of relative risk into absolute risk for a meaningful time period N/A |
| Other analyses | 17 | Report other analyses done—eg analyses of subgroups and interactions, and sensitivity analyses TABLE 1 and FIGURE 1 |
| Discussion | | |
| Key results | 18 | Summarise key results with reference to study objectives PAGE 13 LINES 239-246 (for patients characteristics) and LINES 248-252 (for survival rates) and PAGE 14 LINES 262-273 (for predictors of survival) |
| Limitations | 19 | Discuss limitations of the study, taking into account sources of potential bias or imprecision. Discuss both direction and magnitude of any potential bias PAGE 15 LINES 294-300 |
| Interpretation | 20 | Give a cautious overall interpretation of results considering objectives, limitations, multiplicity of analyses, results from similar studies, and other relevant evidence PAGE 15 LINES 274-280 |
| Generalisability | 21 | Discuss the generalisability (external validity) of the study results PAGE 15 LINES 282-293 |
| Other information | | |
| Funding | 22 | Give the source of funding and the role of the funders for the present study and, if applicable, for the original study on which the present article is based PAGE 18 LINES 359 |

*Give information separately for exposed and unexposed groups.

**Note:** An Explanation and Elaboration article discusses each checklist item and gives methodological background and published examples of transparent reporting. The STROBE checklist is best used in conjunction with this article (freely available on the Web sites of PLoS Medicine at http://www.plosmedicine.org/, Annals of Internal Medicine at http://www.annals.org/, and Epidemiology at http://www.epidem.com/). Information on the STROBE Initiative is available at http://www.strobe-statement.org.

Supplementary Table 1, Additional File 2

Follow-up data

Health status data at follow-up was available for 30 patients, representing approximately 10% of the total study sample; Table 1. Telephone interviews were conducted after a mean of 2 years and 4 months (852 ± 278 days) post-discharge from NER to HSICN (range: 382-1315 days). Overall neurological status (GOS-E) remained unchanged between discharge and follow-up interview (Wilcoxon signed-rank test: z=1.00, p=.317) as only one patient showed an improvement (from GOS-E score 3 to 6). Median BI scores did not differ between time of dis-charge and follow-up interview (Wilcoxon signed-rank test: z=1.47, p=.141) while constant scores as well as improvement and deterioration were observed on an individual basis. The decannulation rate (weaned from TC) at the time of follow-up was about 16%.

Table 1 Patient characteristics: Data for follow-up interview sample

|  | Total |
| --- | --- |
| N (%) | 30 (100) |
| Male, N (%) | 18 (60) |
| Primary diagnosis, N (%) |  |
| Stroke | 17 (57) |
| Hypoxic-ischemic brain injury (HIE) | 7 (23) |
| Traumatic brain injury | 3 (10) |
| Critical illness polyneuropathy/myopathy | 0 (0) |
| Other neurological disorders | 3 (10) |
| Time between discharge and interview, days, mean (SD) | 852 (278) |
| Weaned from MV, N (%) | 0 (0) |
| Weaned from TC, N (%) | 5 (16.6) |
| EQ-5D-5L, self-reported health, Index value | 0.22 |
| EQ-5D-5L, self-rated health, VAS, mean (SD) | 35.2 (21.47) |

Supplementary Table 2, Additional File 3

Extended Cox Regression

Patients with DoC were significantly younger than patients without DoC. Based on this, an extended model was calculated by adding the interaction term of age*DoC additional to the predictor DoC and age. Age was used with centered values for age (Age_c). The results showed that the effect of increasing age in the group without DoC is strongly associated with an increased probability of dying; this association is significant. In the group with DoC, on the other hand, there was a weak effect in the opposite direction, which can be calculated by adding the B coefficients of Age_c and the interaction term Age_c*DoC (*B_DoC_Age_c_* = -0.003). In this group, age therefore had almost no effect on the probability of survival. For the predictor DoC, the presence of DoC was significantly positively associated with the probability of survival in patients of average age (Hazard Ratio = 0.282). However, the interaction term Age_c*DoC showed that the protective effect of DoC becomes weaker with increasing age (Hazard Ratio = 0.952). The influence of DoC on the probability of survival is therefore more pronounced in younger patients, while in older patients age is the dominant factor and the presence of DoC becomes less relevant. The model with the interaction term showed that the effect of DoC on survival depends on age.

Table 2 Extended Cox Regression

| **Variables in the Equation** | | | | | | |
| --- | --- | --- | --- | --- | --- | --- |
|  | B | SE | Wald | df | Sig. | Exp(B) = HR |
| Age_c | .046 | .008 | 37.128 | 1 | <.001 | 1.048 |
| DoC | -1.265 | .412 | 9.423 | 1 | .002 | .282 |
| Age_c*DoC | -.049 | .024 | 4.102 | 1 | .043 | .952 |

HR = Hazard Ratio
